# Ice Pack Test Eased Ptosis in a Patient Presenting with a Possible Oculomotor Nerve Schwannoma: A Case Report

**DOI:** 10.3390/neurolint13040050

**Published:** 2021-10-09

**Authors:** Susanne Buechner, Loredana Capone

**Affiliations:** Department of Neurology/Stroke Unit, General Hospital of Bolzano, Lorenz Boehler Street 5, 39100 Bolzano, Italy; loredana.capone@sabes.it

**Keywords:** ice pack test, oculomotor nerve schwannoma, ptosis, third nerve palsy, myasthenia gravis

## Abstract

A 32-year-old man presented with complete third nerve palsy. Brain magnetic resonance imaging revealed a possible sporadic oculomotor nerve schwannoma in the left cavernous sinus. Interestingly, the patient’s ptosis was fluctuating and eased immediately after application of ice over his eyelid. The ice pack test (IPT) is a simple and fast method that can help diagnose myasthenia gravis (MG) in patients with ptosis. Additional diagnostic investigations (antibody assays and repetitive stimulation) excluded associated MG in the patient. Tumor treatment with Gamma Knife radiosurgery was planned. This case highlights the finding that IPT can also be positive in neurogenic (non-myasthenic) ptosis, but its usefulness in other disorders associated with muscle weakness and fatigability remains questionable.

## 1. Introduction

The ice pack test (IPT) is a simple and fast method that can help diagnose myasthenia gravis (MG) in patients with ptosis [1,2,3]. During the IPT, a bag filled with ice is placed over the drooped eyelid for 2 min. In MG, eyelid drooping improves shortly after the test [4]. We present a case report in which the IPT eased ptosis in a patient presenting with a possible oculomotor nerve schwannoma (ONS). The case is of interest because it shows that a positive IPT result can be obtained in neurogenic (non-myasthenic) ptosis.

## 2. Case Report

A 32-year-old man from Pakistan presented to an ophthalmologist complaining of excessive left-eye lacrimation associated with mild ocular pain. The examination revealed ptosis, limitation of upward, downward, and inward gaze, and mydriasis, and he was diagnosed with complete third nerve palsy. The intraocular pressure and fundus examination were normal. Fluorescein angiography, indocyanine green chorioangiography, and optical coherence tomography were all negative. The patient reported a previous mild left-sided head trauma experienced 1.5 years earlier. He had no other medical history and was not taking medication. There was no family history of metabolic, neurological, or autoimmune disorders.

**Figure 1:**
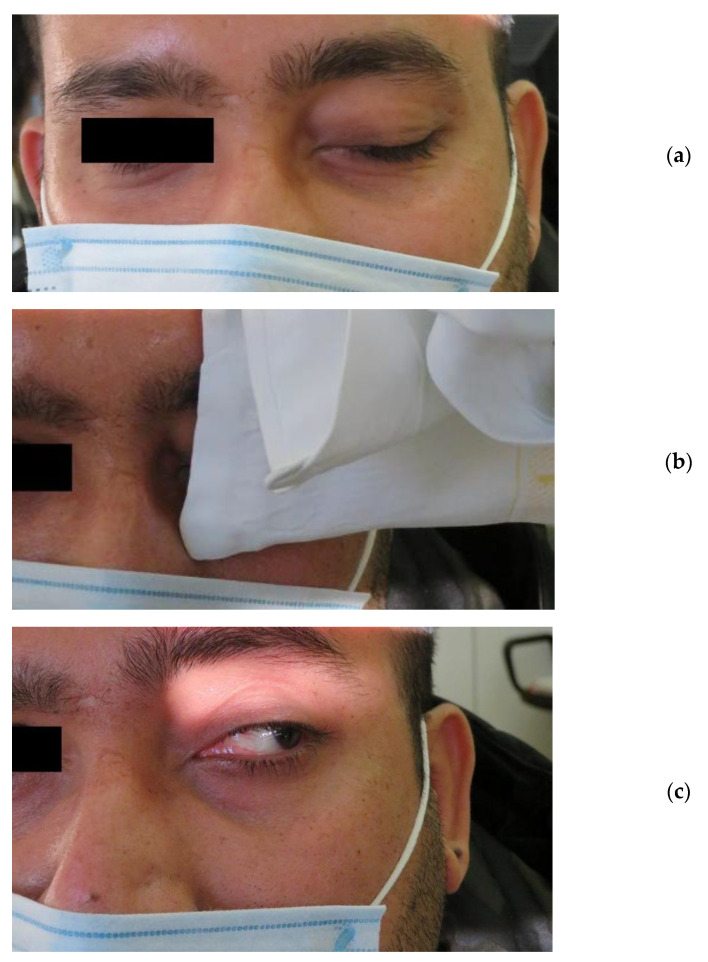
Ice pack application in a patient with neurogenic ptosis. (**a**) Unilateral left-sided ptosis. (**b**) Ice pack over the left drooping eyelid. (**c**) Improvement of the ptosis.

Subsequently, the patient was referred to our neurological clinic where left oculomotor nerve palsy was confirmed. At the neurological examination, no other neurological deficit was found. Interestingly, the patient reported fluctuation of his left-eyelid droop during daytime and significant improvement when washing his face with cold water. The ptosis had developed slowly over the past 4–5 months, and he had suffered from recurrent diplopia. We decided to perform an IPT to exclude MG associated with the oculomotor nerve palsy, which was a definite diagnosis due to the pupillary involvement. We applied an ice pack to the patient’s left eyelid for 2 min, and his ptosis improved significantly (Figure 1a–c). His left eye opened nearly completely, although the improvement was only temporary, lasting just a few minutes. Was it possible that this patient had two neurological conditions, namely an oculomotor nerve palsy and MG?

**Figure 2:**
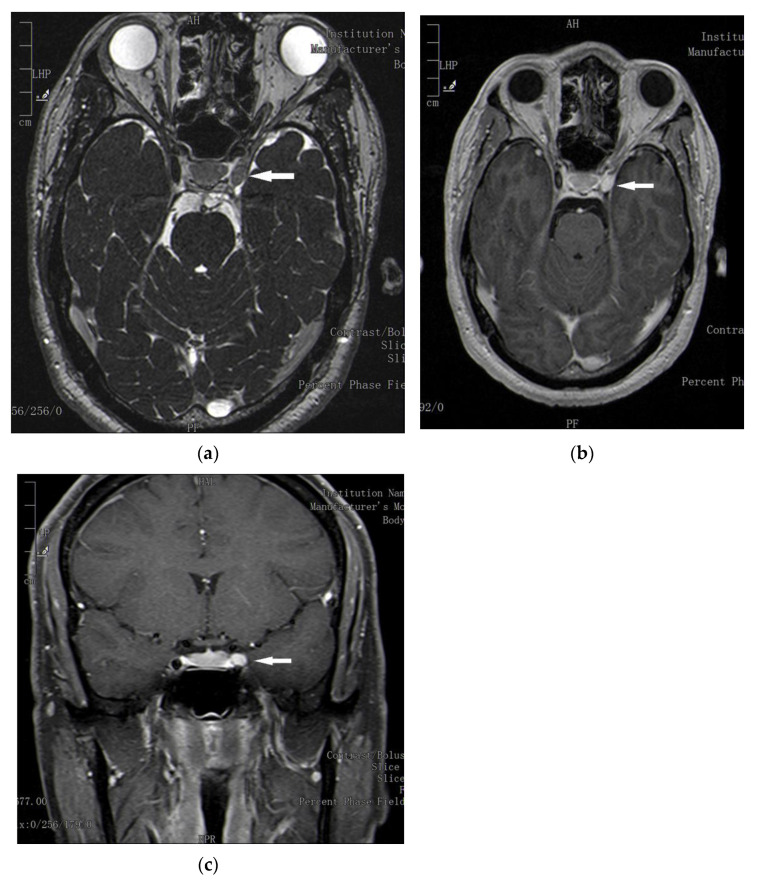
Brain magnetic resonance imaging demonstrating (see respectively white arrow) a possible oculomotor nerve schwannoma in the left sinus cavernous, highly enhanced by gadolinium (Gad). (**a**) T2-weighted axial magnetic resonance imaging (MRI) brain without Gad. (**b**) T1-weighted axial brain MRI with Gad. (**c**) T1-weighted coronal brain MRI with Gad.

Plain and post-contrast brain magnetic resonance imaging (MRI) showed a 10 × 6× 7 mm nodular lesion on the left cavernous sinus lateral wall in continuity with the third cranial nerve, isointense on T2-weighted images and gadolinium enhanced on T1-weighted images. Due to the localization and morphology of the lesion and other radiological characteristics, our neuroradiologists supposed a schwannoma of the left third cranial nerve (Figure 2a–c). It is true that schwannomas reported in the literature are often larger and heterogeneously hyperintense on T2-weighted sequences; however, schwannomas’ appearance at MRI is variable because of the possibility of different histologic patterns [5,6,7,8,9]. Unfortunately, due to the lesion’s small size and localization, our neurosurgeons did not perform stereotactic biopsy; therefore, no tissue for histologic analysis was available. Other tumor lesions, such as pituitary macroadenomas or meningiomas, were excluded by our neuroradiologists. There were also no radiological signs of raised intracranial pressure, vascular ischemia, hemorrhage, or cavernous sinus infection or trauma. As ONS are extremely rare, brain and neck computed tomographic angiography was performed and ruled out a thrombosed aneurysm of the posterior communicating artery and a thrombosis of the left cavernous sinus. The brain MRI angiography had demonstrated a caudal dislocation of the left internal carotid artery, but a Doppler ultrasound study showed completely normal blood flows. Electrodiagnostic testing consisting of repetitive stimulation of the patient’s left facial nerve was not suggestive of a neuromuscular junction disease. The anti-acetylcholine receptor (AChR) antibodies and the antibodies against muscle-specific kinase were negative. There was no evidence of any specific skin lesions on physical examination.

Thus, the final diagnosis, based on clinical and MRI findings and not on histopathological features, was left third nerve palsy caused by a possible sporadic ONS in the left cavernous sinus.

At present, there is no standard treatment for these tumors as they are extremely rare. After an extensive tumorboard discussion, our neurosurgeons decided to refer the patient to a nearby University Hospital for treatment with Gamma Knife radiosurgery (GKRS). This treatment option was chosen due to the small tumor size (<3 cm), the difficult location for surgical approach, and because it can destroy such lesions. Unfortunately, in this case, we could never be sure of the precise nature of this lesion and how the tumor might have progressed, even though we were convinced that the patient was suffering from an ONS. Of course, the brain MRI will be repeated before GKRS treatment. If the tumor might have grown significantly, additional investigations such as a total-body positron-emission tomography scan will be performed, and treatment options will be revaluated.

## 3. Discussion

Schwannomas, also known as neurinomas, are usually benign, slowly growing tumors of peripheral nerves [10] that commonly arise from the Schwann cell layer of a peripheral nerve’s myelin sheath, and account for 6–8% of all intracranial neoplasms [11,12]. Most (approximately 90%) are located on the vestibular branch of the eighth cranial nerve (vestibulocochlear or acoustic nerve). However, schwannomas of the fifth (trigeminal nerve), the seventh (facial nerve), and lower cranial nerves are also quite common [13,14]. A sporadic ONS not associated with neurofibromatosis is extremely rare [12,13]. Kovacs probably described the first case of an isolated ONS during an autopsy in 1927 [15]. To date, there are approximately 60 case reports of ONS in the literature [16,17]. These rare tumors seem to be slightly more frequent in females and arise at a mean age of 35 years [17]. According to the anatomical location, ONS can be divided in cisternal, cisterno-cavernous, and cavernous lesions [18]. However, orbito-cavernous and orbital locations are also possible [17]. The presenting symptoms depend on the tumor’s size and location. Diplopia and headache are very common [17,19], and a certain degree of third nerve palsy is almost always present [20]. To date, there is no standard treatment for ONS. Very small and asymptomatic tumors can simply be followed up, but most cases are managed with surgical resection (total or subtotal). Surgical treatment is nearly always complicated by complete third nerve palsy despite anatomical preservation of the oculomotor nerve, because this nerve is very fragile and susceptible to injury [17,21]. GKRS is an alternative treatment approach, although only a few successfully treated cases have been reported in the literature [22,23]. To preserve the oculomotor nerve function, combined treatment consisting of microsurgery followed by radiosurgery might be the best option.

At the time of writing, the patient’s ONS is planned for GKRS. This treatment strategy was chosen because of the small tumor size. The patient already has a complete third nerve palsy with significant exotropia of his left eye, visible also on brain MRI (Figure 2a), and it is unlikely that the clinical recovery will be complete even if the treatment is successful. In our opinion, the patient was examined in an advanced stage of his oculomotor nerve palsy, and he might have presented late because of the great fluctuation of his symptoms. The patient sought medical attention only when his ptosis became nearly permanent and his left eye presented excessive lacrimation and pain.

We found the fluctuation of the patient’s ptosis, especially with significant improvement under cold water, to be a very interesting symptom, and the positive outcome of the IPT was very surprising. Even when the diagnosis of a possible left thir dnerve schwannoma had already been made, we decided to continue with the diagnostic assessment of MG. Schwannomas grow very slowly, and a degree of clinical fluctuation might be possible in the early stages of the tumor. However, the nearly complete ptosis resolution after IPT was a curious observation. After the exclusion of associated MG, we were still left with the question: Why was the IPT result positive?

MG is an autoimmune disorder in which antibodies interfere with synaptic transmission at the neuromuscular junction [24]. Ocular MG is defined as muscular weakness that remains limited to the eyelids and extraocular muscles, although many patients with ocular MG also have subclinical abnormal neuromuscular transmission in their limb musculature [25]. Pathophysiologically, MG is characterized by a reduction in skeletal muscle postsynaptic AChRs, resulting in a decrease in the end plate potential necessary for action potential generation. The clinical hallmarks of MG are muscle weakness and fatigability, and symptoms are intermittent as they worsen with activity and improve at rest. Ocular MG is characterized by diplopia and/or ptosis, whereas pupillary function is always normal. It is known that local cooling improves neuromuscular transmission [26]; therefore, cold alleviates MG symptoms, including ptosis [4]. It is believed that the cold temperature reduces cholinesterase activity, which increases the availability of acetylcholine at the neuromuscular junction, promoting the efficiency of acetylcholine in eliciting depolarization at the motor end plate [27].

The IPT is a fast, easy, non-invasive, safe, and inexpensive procedure that can be useful in diagnosing suspected ocular MG [1,2,3]. During the IPT, a bag filled with ice is placed over the drooped eyelid for 2 min. In MG, eyelid drooping improves shortly after the test [4]. The IPT result is considered to be positive when the margin reflex distance (MRD) (i.e., the distance between the center of the pupillary light reflex point and the upper eyelid margin) or the palpebral fissure height (PFH) (i.e., the vertical palpebral aperture distance between the upper- and lower-eyelid margins in the pupillary plane), with eyes in the primary position of gaze, improves by >2 mm after the local ice application [2,3,28]. The IPT has a diagnostic accuracy similar to that of single-fiber electromyography in patients with ocular MG [29] and has a high sensitivity and specificity in diagnosing myasthenic ptosis. However, sensitivity and specificity of IPT differ among studies. For example, Natarajan et al. reported a sensitivity of 96% and specificity of 100% [2], whereas Giannacoccaro et al. reported a sensitivity of 86% and a specificity of 79% [29]. To increase the sensitivity of the IPT, Kee et al. proposed a new IPT, particularly useful in patients with mild ptosis, in which the ice is placed on the eyelid after fatigue induction by sustained up-gaze [30].

Usually, IPT is only used in patients who present with ptosis suspected of being related to ocular MG, as an accurate neurological examination is sufficient in differentiating a neuromuscular disease from peripheral nerve or muscle disorders causing ptosis. For example, the clinical features of Horner’s syndrome are mild ptosis, pupillary miosis, apparent enophthalmus, and anhydrosis; therefore, performing an IPT is not indicated. IPT is also unnecessary for patients presenting with ptosis due to neuropathy of the third nerve because the ophthalmoplegia and pupillary involvement already exclude the diagnosis of MG. Indeed, the utility of IPT in other neurological disorders associated with muscle weakness and ptosis remains unclear.

It is not certain why the ptosis of our patient significantly improved after IPT. A mild improvement in ptosis related to oculomotor nerve palsy after IPT also has been observed by Kee et al., although the resulting MRD was significantly larger in MG patients [30]. Thus, improvement in neurogenic ptosis due to cold does not seem to be a completely new and unknown phenomenon. To understand its mechanism, it is useful to review the upper eyelid’s anatomy: the main eyelid elevator is the levator palpebrae superioris muscle (LPSM), which is a skeletal muscle composed of striatal muscular fibers that are mostly under voluntary control [31]. However, our patient did not suffer from myopathy; there was no evidence of LPSM abnormalities due to his previous head trauma. Additionally, myogenic ptosis (and traumatic ptosis) does not improve with cooling [1].

Interestingly, there is also an accessory muscle that contributes to the upper-eyelid elevation called the superior tarsal muscle (STM), also known as Müller’s muscle [31,32]. The STM is a smooth muscle innervated by the sympathetic fibers of the superior division of the oculomotor nerve. Cold exposure increases sympathetic activity [33] and Müller’s muscle contraction contributes to 2 mm of eyelid elevation. Was the cold-induced sympathetic activation of the STM responsible for the improvement in our patient’s ptosis after IPT? We believe that the above-described mechanism of IPT response in MG might also play a role in the cold-related improvement in neurogenic ptosis. Even in healthy individuals, cold decreases the acetylcholinesterase activity at the neuromuscular junction, resulting in increased acetylcholine in the junction and stimulation of muscle contraction.

We are not certain if this is the correct answer to our question posed earlier of why the IPT result was positive, but the good reaction of the eyelid’s elevator muscle might imply that the patient’s third cranial nerve function is not completely lost, and that GKRS treatment might be able to preserve part of the oculomotor function. In addition, the patient’s tumor is not extremely huge; therefore, GKRS treatment might be less aggressive. If the patient’s third cranial nerve function eventually is lost with certainty, then oculomotor nerve reconstruction might be necessary to improve the patient’s quality of life. Isolated ptosis correction is usually contraindicated in patients with oculomotor nerve palsy [28], as this exposes disturbing strabismus that causes diplopia, resulting in headache.

## 4. Conclusions

The IPT has a high specificity and sensitivity in the differential diagnosis of myasthenic ptosis. Its usefulness in other disorders associated with muscle weakness and fatigue remains unclear. This case report, in which the IPT eased ptosis in a patient presenting with a possible ONS, is interesting because it shows that the IPT can give a positive result in neurogenic (non-myasthenic) ptosis. The association of a sporadic ONS with MG is highly improbable. However, in our opinion, diagnostic assessment should always be accurate, and unexpected results require further investigations to search for explanations.

## Abbreviations

AChR	acetylcholine receptor
Gad	gadolinium
GKRS	Gamma Knife radiosurgery
IPT	ice pack test
LPSM	Levator palpebrae superioris muscle
MG	myasthenia gravis
MRD	margin reflex distance
MRI	magnetic resonance imaging
ONS	oculomotor nerve schwannoma
PFH	palpebral fissure height
STM	superior tarsal muscle
